# Hand Book of the Diseases of the Eye

**Published:** 1895-08

**Authors:** 


### HANDBOOK OF DISEASES OF THE EYE. By Dr. A. Eugen Fick, of the University of Zurich. Authorized translation by Dr. Albert B. Hale, of Chicago. In preparation for early issue. The retail price will be from $3.00 to $4.00. Philadelphia: P. Blakiston, Son & Co., 1012 Walnut Street.

